# Calprotectin and SARS-CoV-2: A Brief-Report of the Current Literature

**DOI:** 10.3390/healthcare9080956

**Published:** 2021-07-29

**Authors:** Mauro Giuffrè, Luigi Vetrugno, Stefano Di Bella, Rita Moretti, Debora Berretti, Lory Saveria Crocè

**Affiliations:** 1 Department of Medical, Surgical and Health Sciences, University of Trieste, 34127 Trieste, Italy; GFF.MAURO@GMAIL.COM (M.G.); stefano932@gmail.com (S.D.B.); moretti@units.it (R.M.); lcroce@units.it (L.S.C.); 2 Italian Liver Foundation, 34149 Trieste, Italy; 3 Department of Medicine, Anesthesia and Intensive Care Clinic, University of Udine, 33100 Udine, Italy; 4 Debora Berretti—Gastroenterology Unit, Azienda Sanitaria Universitaria Friuli Centrale, 33100 Udine, Italy; debora.berretti@asufc.sanita.fvg.it

**Keywords:** fecal calprotectin, calprotectin, COVID-19, SARS-CoV-2, IBD

## Abstract

In late December 2019, a novel coronavirus (lately referred to as SARS-CoV-2) spread in the city of Wuhan, China, causing an outbreak of unusual viral pneumonia. In many people, the disease is mild and self-limiting, but in a considerable number of patients, the disease may present more severe or even fatal. Therefore, determining which patients are at higher risk of developing a more severe disease is critical. Some studies have been focused on serum and fecal calprotectin to evaluate COVID-19 disease progression and possible complications. Some assumptions can be made: (1) serum calprotectin may efficiently predict the prognosis of COVID-19 patients; (2) fecal calprotectin may appear high in COVID-19 patients due to the double hit mechanism to the intestine (inflammatory and ischemic); (3) a relationship between the complement system and neutrophil activation contributes to the procoagulant status seen in COVID-19 patients; (4) some patients may develop severe gastro-intestinal complications and fecal calprotectin can be used to monitor intestinal disease activity levels.

## 1. Introduction

In late December 2019, a novel coronavirus spread in the city of Wuhan, China, causing an outbreak of unusual viral pneumonia [1]. These patients showed symptoms of viral pneumonia, including fever, cough, and in more severe cases, dyspnea with bilateral lung infiltration requiring patients′ admission to intensive care units (ICU) [1]. The cause remained unknown until 9 January 2020, when the genome sequence of the virus was sequenced and made available to the scientific community [1]. Initially referred to as 2019-nCoV and later as severe acute respiratory syndrome coronavirus 2 (SARS-CoV-2), it was branded COVID-19 by the World Health Organization (WHO) in February 2020 [2].

In many people, the disease is mild and self-limiting, but in a considerable number of patients, the disease may present more severe or even fatal. At the moment of writing this report, almost 123 million people have been infected with SARS-CoV-2, with 2.7 million deaths worldwide by COVID-19. Therefore, determining which patients are at higher risk of developing a more severe disease is critical. For example, patients with certain risk factors such as older age, diabetes, obesity, and coronary artery disease are more likely to be admitted to the hospital [3]. Moreover, several risk scores accounting for risk factors and laboratory values have been proposed and none of them have performed efficiently in predicting COVID-19 progression or have been externally validated with excellent results [4,5,6], which is easy to understand considering that the pathogenesis of COVID-19 in humans has not yet been fully elucidated. Leukopenia is a common finding of peripheral blood of these patients [5]. However, tissue and organ analysis revealed a significant neutrophilic infiltrate. Enhanced neutrophil infiltration, alongside the release of neutrophil extracellular traps, is likely to contribute to the organ damage seen in COVID-19 [5]. Neutrophils’ role in COVID-19 has been increasingly investigated, together with the role of surrogate markers of neutrophilic activation such as calprotectin. In fact, some studies have focused on calprotectin concentrations in serum and fecal samples of patients with COVID-19 in order to study disease progression and possible complications [7]. The aim of this narrative brief-report is to summarize the current evidence on calprotectin in COVID-19. The current literature search was conducted to identify recent papers on this subject on the MEDLINE database using the following keywords: “COVID-19”, “calprotectin”, “S100A8/A9”, “serum calprotectin”, and “fecal calprotectin” from 1 December 2019 to 30 June 2021. Essentially, we evaluated studies that included patients with a minimum age of 18 years that tested positive by RT-PCR to SARS-CoV-2, whose level of serum or fecal calprotectin were measured regardless of the clinical setting.

## 2. What Is Calprotectin?

Calprotectin belongs to the family of the S-100 proteins, first isolated from blood leukocytes in 1980 [8]. Calprotectin constitutes as much as 60% of the soluble protein content of the cytosol of neutrophils (despite being present in lower concentrations in macrophages, granulocytes, and monocytes) and is secreted during the inflammatory response. Human calprotectin is a 24 kDa [9] dimer formed by the two protein monomers of S100-A8 (10.8 kDa) and S100-A9 (13.2 kDa). Occasionally, the two monomers can bind non-covalently to form 48 kDa tetramers [9]. S100-A8/A9 genes are located on chromosome 1q21 [10].

Calprotectin is a metal-binding protein with a high affinity especially to calcium but also to zinc, iron, and manganese [11]. Each monomer has calcium-binding sites and can bind two calcium ions [12]. Calcium-binding induces conformational changes that increase calprotectin affinity for other transition metals [9,11,13].

Upon neutrophil activation or death, calprotectin is released to the extracellular environment, where it can exert its function. In particular, calprotectin is known to have direct bactericidal and pseudo-cytotoxic properties that arise from its active sequestration of zinc and manganese ions, which prevent its use in pathogens’ metabolic and replicative processes [9,13]. Given its relatively small size, easy tissue-to-blood diffusion, and resistance to enzymatic degradation, calprotectin is a sensitive marker of neutrophil activation [14,15], with a reported half-life of five hours [16]. Furthermore, calprotectin is an endogenous ligand of toll-like receptor 4 (TLR-4), triggers the inflammatory response via tumor-necrosis factor α, and is a mediator on the rapid rearrangement of the cytoskeleton, which is essential for cell migration during the immune response [17]. Calprotectin release also weakens cellular adhesion, favoring leukocyte extravasation [17].

Serum levels are usually reported below 1 µg/L in healthy individuals; however, serum concentration may increase by 100 times in inflammation. On the other hand, fecal calprotectin (FC) is released by neutrophils that reach the intestinal membrane during active inflammation and is then excreted in the stool [17]. It is still unclear whether or not there is a quantitative correlation between serum and fecal calprotectin [17].

## 3. Serum Calprotectin and COVID-19

As stated in the previous paragraph, calprotectin is predominantly restricted to the intracellular compartment of neutrophils, which are the most abundant human innate immune effector cells, possessing a broad variety of antimicrobial substances that are stored in specialized granules. Given that these substances can also damage host tissues, their release is strictly regulated through three major processes: phagocytosis, degranulation, and the release of neutrophil extracellular traps (NETs) [18]. NETs are large extracellular web-like structures principally composed of cytosolic and granule proteins (including calprotectin) that are assembled over a scaffold of decondensed chromatin. NETs are extremely efficient in trapping, neutralizing, and killing bacteria, viruses, fungi, and parasites [18,19]. In addition, elevated blood levels of NETs and neutrophil-derived calprotectin were associated with a higher risk of morbid thrombotic events in COVID-19 patients despite prophylactic anticoagulation [7,20,21].

In addition to possible pathogenic mechanisms, serum calprotectin does not need de novo synthesis, thus offering a decisive kinetic advantage as a first sign of severe inflammation in contrast to other routinely measured serum biomarkers such as CRP or procalcitonin (PCT). For example, Silvin et al. found that patients with severe COVID-19 exhibited exponentially higher blood calprotectin if compared to patients with more moderate disease or controls [22]. In addition, calprotectin concentrations correlated with neutrophil count (R = 0.62, *p* < 0.001), plasma fibrinogen (R = 0.76, *p* < 0.001) and D-Dimer (R = 0.64, *p* < 0.001) [22]. Similar data were reported by Shi et al., who analyzed sera obtained from 172 patients [23]. In particular, they found that calprotectin levels were significantly higher in those individuals who required mechanical ventilation at any point during their hospitalization (8039 vs. 3365 ng/mL, *p* < 0.001) [23]. Moreover, calprotectin was found to correlate with C-reactive protein (CRP) (R = 0.44, *p* < 0.001), neutrophil count (R = 0.50, *p* < 0.001), ferritin (R = 0.31, *p* < 0.001), lactate dehydrogenase (R = 0.52, *p* < 0.001), and platelet count (R = 0.39, *p* < 0.001), and to discriminate between patients that required mechanical ventilation and those who did not, with an area under the receiver operating characteristic (AUROC) of 0.794 [23]. However, the authors did not provide calprotectin cut-offs and their respective discriminative metrics [23]. Furthermore, Cherubini et al. found that patients with SARS-CoV-2 had higher average plasma calprotectin levels (352.3 ng/mL) if compared to (1) patients with symptoms but negative nasopharyngeal PCR-testing (177.2 ng/mL) or (3) individuals without symptoms and negative nasopharyngeal PCR-testing (45.3 ng/mL). The authors reported that calprotectin can discriminate between symptomatic patients (COVID-positive vs. COVID-negative) with an AUROC of 0.72 and reported the cut-off 131.3 as being the most performant, with a specificity of 70.77% and a sensitivity of 69.49% [24].

Bauer et al. reported that serum calprotectin had the best discriminative ability to predict ICU admission (AUROC 0.70, 95% C.I. 0.42–0.99) and multi-organ failure within 72 h (AUROC 0.87, 95% C.I. 0.63–1) if compared to other commonly employed biomarkers (i.e., lactate, CRP, PCT) [25]. Comparable results were reported by Chen et al. in terms of serum calprotectin prediction of ICU admission and subsequent death [26]. In particular, the authors highlighted that patients with higher serum calprotectin had a 13-fold risk of death at 60 days from hospital admission [26]. In a recent case series of 66 patients, De Guardiana-Romualdo et al. reported that hospitalized COVID-19 patients who did not survive the infection had two-fold the median values of serum calprotectin than those who survived [27].

Current findings in terms of serum calprotectin and COVID-19 are summarized in Table 1.

## 4. Fecal Calprotectin and COVID-19

Previous studies indicate that SARS-CoV-2 binds to intestinal epithelial cells via specific receptors such as angiotensin-converting enzyme-2 (ACE-2) and the transmembrane serine protease-2 [28,29]. The virus can infect intestinal epithelial cells, promoting acute inflammation characterized by infiltration of neutrophils and macrophages, thus the hypothesis of increased fecal calprotectin as a surrogate of intestinal infection [30]. Gastrointestinal symptoms are present in up to 28% of patients with COVID-19 [31,32,33] and fecal SARS-CoV-2-RNA was detected in approximately 50% of positive individuals [32,33,34], which persists after clearance from respiratory samples [35], with a mean fecal shedding of 17 days [36]. Current findings in terms of FC and COVID-19 are summarized in Table 2.

Effenberger et al. analyzed 40 patients with COVID-19 admitted at the University Hospital of Innsbruck (Austria) [30]. Patients with acute diarrhea showed a higher FC level if compared to patients without diarrhea (123.2 vs. 17.3 µg/g, *p* < 0.001) [30]. FC concentration correlates with interleukin-6 but not to other markers of inflammation such as CRP [31]. Notably, viral RNA was not detected in stools from patients with ongoing diarrhea, and no relation was found between SARS-CoV-2 RNA and FC [30].

Zerbato et al. enrolled 51 consecutive adults with SARS-CoV-2 pneumonia. The authors did not detect any differences in FC concentrations between patients with and without diarrhea. However, the patients with SARS-CoV-2 RNA detected in fecal samples had higher FC (74 vs. 39 mg/kg, *p* < 0.001), lower neutrophil counts (5550 vs. 4390 cell/µL, *p* < 0.035), and higher D-Dimer (723 vs. 580 ng/mLFEU) [40]. On the contrary, Britton et al. did not find any correlation or difference in FC concentrations between patients stratified by gastrointestinal symptoms or detection of fecal viral-RNA [38].

Ojetti et al. conducted an observational study of 65 patients admitted to the Emergency Department of the Gemelli University Hospital (Rome, Italy) [39]. Elevated FC levels (>50 µg/g) were found in 29.2% of patients. Among these, 57.9% showed radiological findings compatible with COVID-19, whereas only 10.9% of patients in the group with normal FC showed radiological anomalies [41]. In particular, patients with radiological interstitial pneumonia had higher FC if compared to patients without anomalies (71.3 vs. 11.9 µg/g, *p* < 0.001). In addition, patients with normal FC were younger (33 vs. 56 years old, *p* = 0.0024) and mostly men (87% vs. 52.6%) [41]. Also, patients with elevated FC were more likely to have gastrointestinal symptoms (47.4% vs 15.2%, *p* = 0.006) [41].

### Fecal Calprotectin and Mesenteric Ischemia in COVID-19 Patients

According to a few studies, FC can be slightly elevated in patients with ischemic bowel disease [39,42]. Currently, thirteen cases of acute mesenteric ischemia in COVID-19 patients have been reported [43]; however, FC has not been evaluated in any of the aforementioned cases.

Interestingly, Giuffrè et al. enrolled 25 consecutive patients, of which 84% showed increased FC despite being asymptomatic for gastrointestinal symptoms. The authors did not detect any significant correlation between FC and CRP. However, they found a strong positive correlation between FC and D-Dimer (R = 0.745, *p* < 0.001) [37]. Besides, two patients with particularly high FC developed spontaneous intestinal perforation [37,44]. The authors hypothesized that the mechanism behind intestinal perforation was related to a thrombosis localized to the gut and that FC increase is related to virus-related inflammation and to thrombosis-induced ischemia, as showed by gross pathology [45].

## 5. Conclusions

Unfortunately, the evidence on the role of calprotectin in COVID-19 is only in its infancy. However, some assumptions can be made: (1) serum calprotectin may efficiently predict the prognosis of COVID-19 patients; (2) FC may appear high in COVID-19 patients due to the double hit mechanism to the intestine (inflammatory and ischemic); (3) a relationship between the complement system and neutrophil activation contributes to the procoagulant status seen in COVID-19 patients; (4) some patients may develop severe gastro-intestinal complications and FC can be used to monitor intestinal disease activity levels. Nevertheless, more studies are required to further define the role of calprotectin in COVID-19 patients.

## Figures and Tables

**Table 1:** Summary of current findings in terms of serum calprotectin and COVID-19. ICU: intensive care unit; CRP: C-reactive proteins; AUROC: area under the receiver operating characteristic.

Authors, Country	Design	Sample Size	Primary Results/Conclusions
Chen et al. [27] (July, 2020) Wuhan (China)	Retrospective	Total, *n* = 121	Mean Calprotectin Concentrations: ICU, 9220 ng/mL vs. non-ICU, 7800 ng/mL (*p* = 0.0001).
		ICU, *n* = 40	Serum calprotectin can discriminate with an AUROC of 0.86 and a cut-off of 6195 ng/mL (sensitivity 85%, specificity 82.7%) ICU admission. Also, patients with serum calprotectin > 6195 ng/mL had a 13-fold risk of death at 60 days from hospital admission.
		Non-ICU, *n* = 81	
Shi et al. [24] (July, 2020) Michigan (USA)	Cohort	Total, *n* = 172	Mean Calprotectin Concentrations: patients who needed ventilation, 8039 ng/mL vs. those who did not, 3365 ng/mL (*p* < 0.0001).
		Room air group, *n* = 41	Calprotectin levels were significantly higher in those individuals who required mechanical ventilation at any point during their hospitalization. Serum calprotectin could discriminate between patients that required mechanical ventilation and those who did not, with an AUROC of 0.794.
		Non-invasive oxygen, *n* = 71	
		Invasive ventilation, *n* = 60	
De Guadiana-Romualdo et al. [28] (August, 2020) Cartagena (Spain)	Case Series	Total, *n* = 66	Mean Calprotectin Concentrations: survivors, 3540 ng/mL vs. non-survivors, 7900 ng/mL (*p* < 0.001).
		Survivors, *n* = 8	Serum calprotectin positively correlated with other inflammation markers and was significantly higher in non-survivors, thus highlighting a possible prognostic role in COVID-19 patients.
		Non-Survivors, *n* = 58	
Silvin et al. [23] (August, 2020) Villejuif (France)	Cohort	Total, *n* = 158	Mean Calprotectin Concentrations: severe, 4983 ng/mL vs. non-severe 985 ng/mL (*p* < 0.0001).
		Severe, *n* = 50	Patients with more severe COVID-19 exhibited exponentially higher serum calprotectin if compared to patients with more moderate disease or controls. Serum calprotectin can discriminate between severe and non-severe disease with an AUROC of 0.959.
		Non-Severe, *n* = 39	
		Controls, *n* = 86	
Bauer et al. [26] (November, 2020) Berlin, Germany	Cohort	Total, *n* = 19	Mean Calprotectin Concentrations: ICU, 3770 ng/mL vs. non-ICU, 2080 ng/mL (*p* = 0.15).
		ICU, *n* = 8	Serum calprotectin had the best discriminative ability to predict ICU admission (AUROC 0.70, 95% C.I. 0.42–0.99) and multi-organ failure within 72 h (AUROC 0.87, 95% C.I. 0.63–1) if compared to other commonly employed biomarkers.
		Non-ICU, *n* = 11	
Cherubini et al. [25] (May, 2021) Rome (Italy)	Cohort	Total, *n* = 195	Mean Calprotectin Concentrations: hospitalized patients with positive RT-PCR, 352.3 ng/mL vs. patients with symptoms but negative nasopharyngeal RT-PCR, 177.2 ng/mL vs. individuals without symptoms and negative nasopharyngeal RT-PCR (45.3 ng/mL).
		Hospitalized Patients with positive RT-PCR, *n* = 65	Calprotectin can discriminate between symptomatic patients (COVID-positive vs. COVID-negative) with an AUROC of 0.72 and reported the cut-off 131.3 ng/mL, being the most performant with a specificity of 70.77% and a sensitivity of 69.49%
		Hospitalized Patients with negative RT-PCR, *n* = 59	
		Healthy individuals screened with negative RT-PCR, *n* = 71	

**Table 2:** Summary of current findings in terms of fecal calprotectin and COVID-19. CRP: C-reactive proteins; IL-6: interleukin-6; FC: fecal calprotectin.

Authors, Country	Design	Sample Size	Primary Results/Conclusions
Effenberger et al. [31] (August, 2020) Innstruk (Austria)	Cohort	Total, *n* = 40	Mean Calprotectin Concentrations: patients with diarrhea 80.2 mg/kg vs. patients without diarrhea 17.3 mg/kg.
		Patients with diarrhea, *n* = 22	Patients with acute diarrhea showed higher FC level if compared to patients without diarrhea. FC concentration correlates with IL-6 but not to other markers of inflammation such as CRP. Viral RNA was not detected in stools from patients with ongoing diarrhea, and no relation was found between SARS-CoV-2 RNA and FC.
		Patients without diarrhea, *n* = 18	
Giuffrè et al. [37] (August, 2020) Trieste, Italy	Cohort	Total, *n* = 25	Approximately, 84% of patients showed increased FC despite being asymptomatic for gastrointestinal symptoms. Two patients with particularly high FC developed spontaneous intestinal perforation.
Britton et al. [38] (September, 2020) New York (USA)	Retrospective	Total, *n* = 43	SARS-CoV-2 RNA was seen in stools of 41% of patients, being slightly more prevalent in patients with diarrhea.FC did not correlate withgastrointestinal symptoms or viral level detected.
Ojetti et al. [39] (November, 2020) Rome (Italy)	Cohort	Total, *n* = 65	Mean Calprotectin Concentrations: patients with radiological interstitial pneumonia had higher FC if compared to patients without anomalies (71.3 vs. 11.9 µg/g, *p* < 0.001).
			Patients with normal FC were younger (33 vs. 56 years old, *p* = 0.0024) and mostly men (87% vs. 52.6%). Also, patients with elevated FC were more likely to have gastrointestinal symptoms (47.4% vs 15.2%, *p* = 0.006).
Zerbato et al. [38] (June, 2021) Trieste, Italy	Cohort	Total, *n* = 51	The authors did not detect any differences in FC concentrations between patients with and without diarrhea. However, the patients with SARS-CoV-2 RNA detection in fecal samples had higher FC (74 vs. 39 mg/kg, *p* < 0.001), lower neutrophil counts (5550 vs. 4390 cell/µL, *p* < 0.035), higher D-Dimer (723 vs. 580 ng/mLFEU).

## Data Availability

Not applicable.
